# Investigating the specific core genetic-and-epigenetic networks of cellular mechanisms involved in human aging in peripheral blood mononuclear cells

**DOI:** 10.18632/oncotarget.7388

**Published:** 2016-02-14

**Authors:** Cheng-Wei Li, Wen-Hsin Wang, Bor-Sen Chen

**Affiliations:** ^1^ Laboratory of Control and Systems Biology, Department of Electrical Engineering, National Tsing Hua University, Hsinchu, Taiwan

**Keywords:** human aging, genetic-and-epigenetic network, anti-ageing drugs, microRNAs, DNA methylation, Gerotarget

## Abstract

Aging is an inevitable part of life for humans, and slowing down the aging process has become a main focus of human endeavor. Here, we applied a systems biology approach to construct protein-protein interaction networks, gene regulatory networks, and epigenetic networks, i.e. genetic and epigenetic networks (GENs), of elderly individuals and young controls. We then compared these GENs to extract aging mechanisms using microarray data in peripheral blood mononuclear cells, microRNA (miRNA) data, and database mining.

The core GENs of elderly individuals and young controls were obtained by applying principal network projection to GENs based on Principal Component Analysis. By comparing the core networks, we identified that to overcome the accumulated mutation of genes in the aging process the transcription factor JUN can be activated by stress signals, including the MAPK signaling, T-cell receptor signaling, and neurotrophin signaling pathways through DNA methylation of BTG3, G0S2, and AP2B1 and the regulations of mir-223 let-7d, and mir-130a. We also address the aging mechanisms in old men and women. Furthermore, we proposed that drugs designed to target these DNA methylated genes or miRNAs may delay aging. A multiple drug combination comprising phenylalanine, cholesterol, and palbociclib was finally designed for delaying the aging process.

## INTRODUCTION

Complex biological processes such as aging do not result from an isolated event in a single protein, chemical, enzyme, or individual cell type. Rather, such processes occur as the coordinated result of interacting cell types and tissues, as well as alterations in gene regulation and expression, signaling pathways, and biological networks. It is the combined and synergistic effects of the individual parts that lead to aging [1]. Aging is a complex process comprising a wide variety of interconnected features and effects, such as a progressive functional decline, gradual deterioration of physiological function, and decrease in fertility and viability. Deterioration of physical health is the principal factor associated with aging [2]. Although aging is inevitable, understanding the underlying molecular mechanisms can help to retard the process. This is a key goal of aging research, in addition to designing drug targets to combat the aging process.

Several aging-related studies have been conducted in a variety of animals in the last three decades. For example, basic research conducted in model organisms, particularly *C. elegans*, identified a number of single-gene mutations that confer increased lifespan phenotypes. In addition, genome-wide expression studies have revealed distinct expression profiles associated with aging [3-5]. Jurk *et al.* demonstrated that chronic, and progressive low-grade inflammation induced by knockout of the nfkb1 subunit of the transcription factor NF-κB induces premature aging in mice [6]. However, this study merely demonstrates the effect of a single event, which does not adequately explain the underlying aging mechanisms.

MicroRNAs (miRNAs) are endogenous small RNAs that regulate gene expression mainly at the posttranscriptional level, and have gained increasing attention in the last decade [7, 8]. A single miRNA can target multiple genes, and multiple miRNAs can also target the same gene [9]. The miRNAs involved in aging and lifespan determination have been studied extensively in the last several years. For instance, Liang *et al.* demonstrated up-regulation of miRNA expression with concomitant inverse down-regulation of target genes with aging [10]. Additionally, Yang *et al.* demonstrated in *C. elegans*, that a mir-34 mutant led to an extended lifespan [11]. Understanding how miRNAs associate with aging-related biological processes and pathways can provide deeper insight into the mechanisms of aging.

DNA methylation patterns are shaped by two opposing processes of adding and removing a methyl group at position five of cytosine in DNA [12]. Age-associated alterations in DNA methylation are commonly grouped into a phenomenon known as “epigenetic drift,” which is characterized by gradual extensive demethylation of the genome and hypermethylation of a number of promoter-associated CpG islands [13-15]. Hypermethylation of CpG islands in gene promoters induces gene silencing and alters gene expression. Aging induced DNA methylation has been studied extensively in the last several years. Meaghan *et al.* found that healthy human aging occurs throughout the lifetime. The authors discussed the dynamics of DNA methylation as well as how the interactions between genomics, the environment, and epigenomics influence aging rates [16].

Protein-protein interactions (PPIs) are crucial for signal transduction in biological processes. Therefore, compiling PPIs provides new insight into protein function [17-19]. PPIs have revealed global topological and dynamic features related to well-understood biological properties [20]. This indicates that studying PPIs will allow further understanding of disease mechanisms at a systematic level [21-23]. Wang *et al.* identified *C. albicans*-zebrafish interspecies PPIs and used this information to highlight the association between *C. albicans* pathogenesis and the zebrafish redox process, indicating that redox status is critical in the battle between the host and pathogen [24]. Therefore, PPIs can be an effective way to study the complex biological processes involved in aging.

Transcription factors (TFs) are the main regulators of DNA transcription. Thus, knowing the genes that are targeted by a specific TF is of utmost importance for understanding developmental processes, the cellular stress response, or disease etiology [25, 26]. Recently, Tu and Chen constructed an aging network of TF-gene interactions in *Homo sapiens* [27]. This network of regulator-gene interactions describes potential pathways that yeast cells can use to regulate global gene expression programs. Additionally, Tu *et al.* indicated that the network robustness and response ability of dynamic gene regulatory networks (GRN) play a key role in the aging process [28]. Accordingly, in the current study, PPIs, GRNs, and miRNA regulation were used to construct genetic and epigenetic networks (GENs) in order to understand the molecular mechanisms of human aging.

Principal component analysis (PCA) is a multivariate technique that analyzes a data table in which observations are described by several inter-correlated quantitative dependent variables. The goal is to extract the most important information from the data table. PCA is probably the most popular multivariate statistical technique used in all scientific disciplines. Here, the principal network projection (PNP) method based on PCA was utilized to obtain the core GENs for young and old women and males.

We analyzed the GENs of human aging using a regression model and big data mining. Based on the PPI candidate, TF-gene regulation, and miRNA regulation databases, as well as the gene expression profiles of young and old peripheral blood mononuclear cell samples, two GENs with quantitative regulatory abilities for young and old individuals were constructed. By applying the PNP method, we obtained the core proteins, TFs, target genes, and miRNAs for each of the core GENs in young and old individuals. The intersection of young and old GENs is called the common core GEN, and the distinction between young and old GENs is referred to as the specific core GEN. We garnered insight into the mechanism of human aging by investigating the specific core GEN of old individuals. We observed that these specific core proteins, TFs, target genes, miRNAs, and DNA methylated proteins are involved in aging-associated cellular mechanisms, suggesting that drug therapy to delay human aging can be designed against these targets. In addition, we also investigated gender differences in human aging based on specific core GENs as well as information obtained from the analysis of peripheral blood mononuclear cells.

Overall, this study provides new age-related molecular drug targets as well as unravels the molecular mechanisms of gender-specific changes in the human aging process.

Analysis of the specific core GEN of old individuals demonstrated that inhibition of three key genes (*FLNB*, *CDK4*, and *ZNF274*) mitigated the dysregulation of MAPK, T-cell receptor, and neurotrophin signaling pathways as well as aberrant cell cycle and apoptosis regulation. The specific core GEN of old men and revealed differences in biological regulation during aging. In women, inhibition of *TAOK3* and *TRAF6* genes circumvented dysregulation of the MAPK and Toll-like receptor signaling pathways as well as immune, proliferative, and metabolic dysfunction. However, in men, inhibition of *STMN1* and *LRRFIP2* could not overcome dysregulation of the MAPK and Wnt signaling pathways or aberrant cell cycle and apoptosis regulation, thus resulting in cancer. Based on these findings, we conclude that the MAPK signaling pathway plays the most important role in the human aging process.

## RESULTS

### Molecular mechanisms of human aging in the specific core GENs of elderly men and women

**Figure 1:**
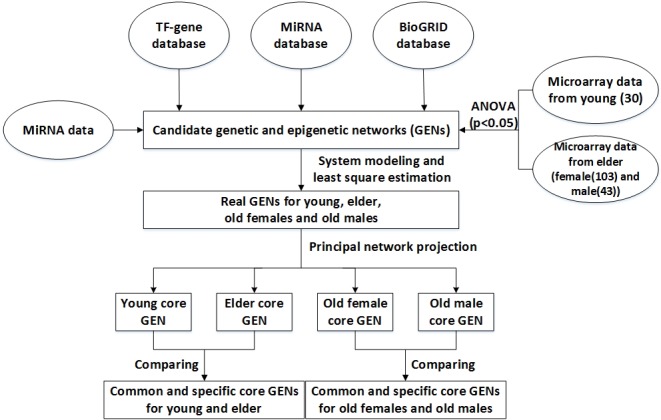
Flowchart for constructing the common and specific core GENs for human aging Microarray data of young and elderly samples, miRNA data, and miRNA, TF-gene, and BioGRID databases were searched to construct candidate GENs, which consisted of interactive candidate GRN, PPIN, and miRNA regulation network. False positives of candidate GENs were then pruned for constructing real GENs of young and old men and women by system modeling and least square estimation. The core GENs were obtained by PNP. The intersection of young and elderly core GENs is called a common core GEN. The distinction between young and elderly core GENs are called young and/or elderly specific core GENs. We investigated different molecular mechanisms between the young and elderly by their specific core GENs. We further investigated the gender-specific aging mechanisms in old women and males.

**Figure 2:**
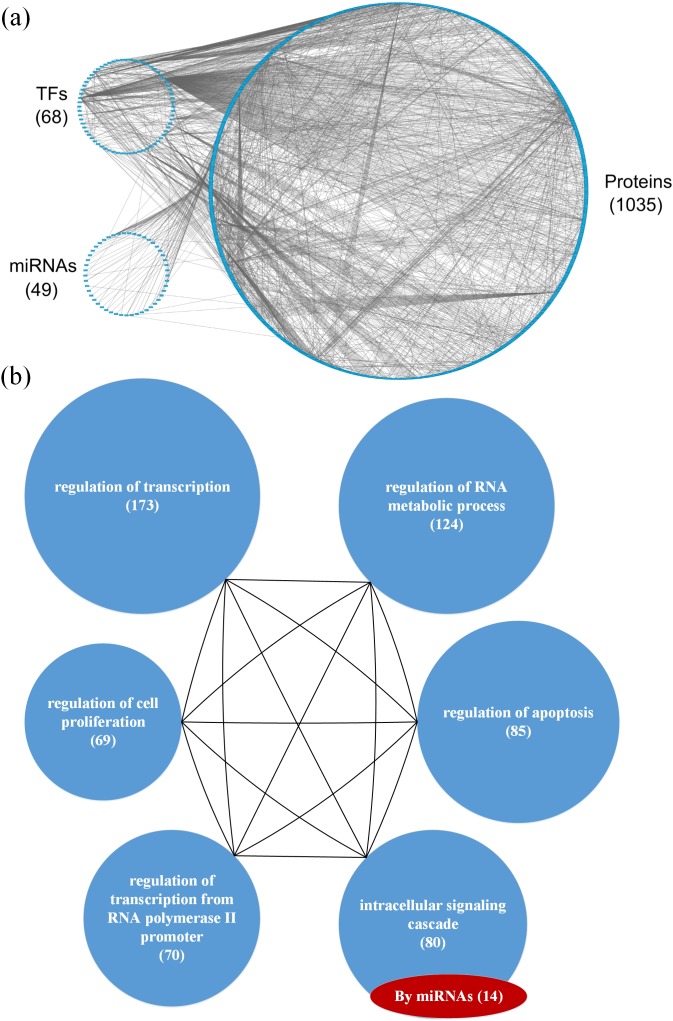
(a) The GEN of young individuals and (b) the associated functional network (a) The upper left circle represents the TFs (68), the lower left circle represents the miRNAs (49), and the right circle represents the proteins (1035) for the GEN of young individuals. Links within the “proteins” circle represent PPIs, links between the “TFs” circle and the “proteins” circle represent regulation of TFs on target genes, and links between the “miRNAs” circle and the “proteins” circle represent regulation of miRNAs on target genes in the GEN of young individuals. (b) In the functional network of the GEN of young individuals, the first six enriched GO terms (biological processes) are regulation of transcription, regulation of RNA metabolic process, regulation of apoptosis, intracellular signaling cascade, regulation of transcription from RNA polymerase II promoter, and regulation of cell proliferation. The numbers in parentheses represents the number of proteins involved in each function.

**Figure 3:**
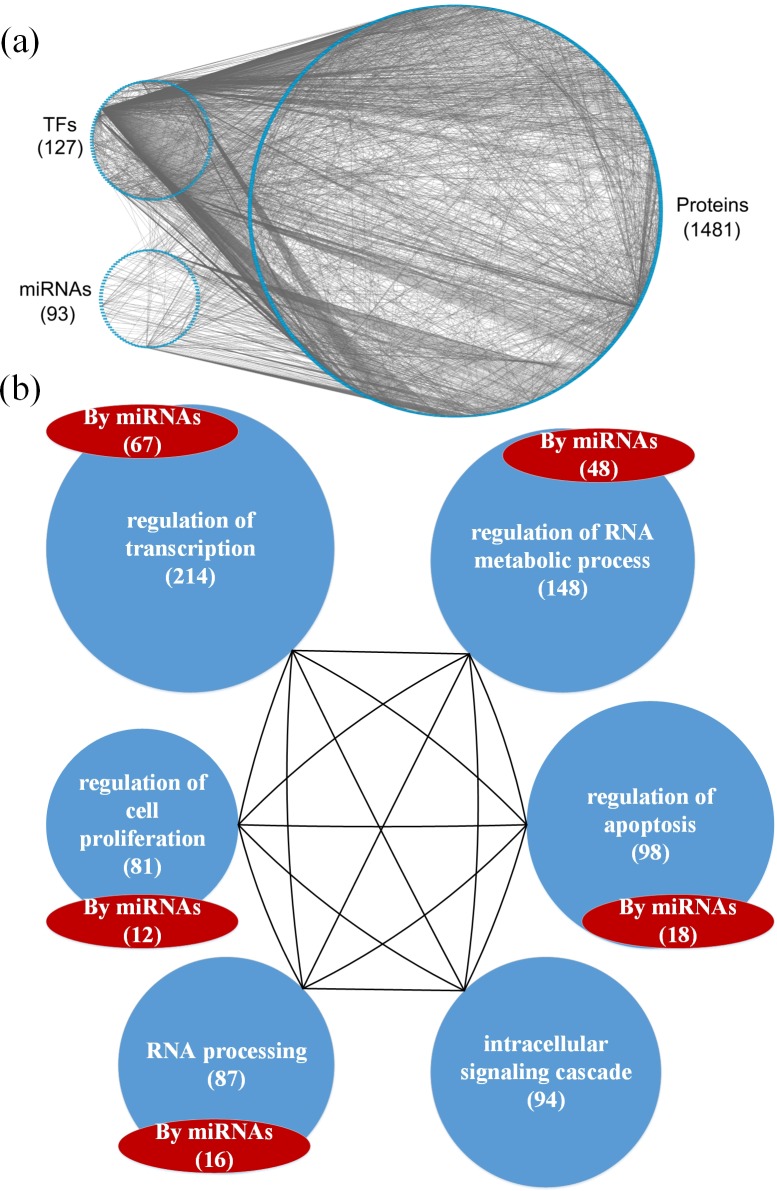
**a.** The GEN of elderly individuals, and **b.** the associated functional network **a.** The upper left circle represents the TFs (127), the lower left circle represents the miRNAs (93), and the right circle represents the proteins (1481) for the GEN of elderly individuals. Links within the “proteins” circle represent PPIs, links between the “TFs” circle and the “proteins” circle represent regulation of TFs on target genes, and links between the “miRNAs” circle and the “proteins” circle represent regulation of miRNAs on target genes in the GEN of elderly individuals. **b.** In the functional network of the GEN of elderly individuals, the first six enriched GO terms (biological processes) are regulation of transcription, regulation of RNA metabolic process, regulation of apoptosis, intracellular signaling cascade, RNA processing, and regulation of cell proliferation. The numbers in parentheses represent the number of proteins involved in each function.

**Figure 4:**
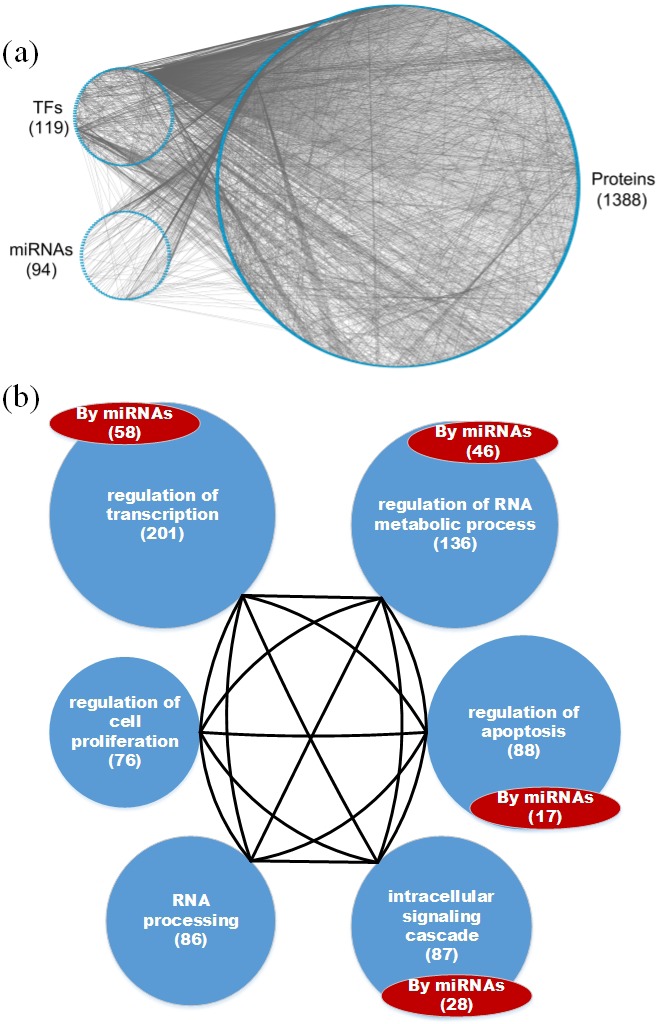
**a.** The GEN of old women and **b.** the associated functional network **a.** The upper left circle represents the TFs (119), the lower left circle represents the miRNAs (94), and the big right circle represents the proteins (1388) for the GEN of old women. Links within the “proteins” circle represent PPIs, links between the “TFs” circle and the “proteins” circle represent regulation of TFs on target genes, and links between the “miRNAs” circle and the “proteins” circle represent regulation of miRNAs on target genes in the GEN of old women. **b.** In the functional network of the GEN of old women, the first six enriched GO terms (biological processes) are regulation of transcription, regulation of RNA metabolic process, regulation of apoptosis, intracellular signaling cascade, RNA processing, and regulation of cell proliferation. The number in parentheses represents the number of proteins involved in each function.

**Figure 5:**
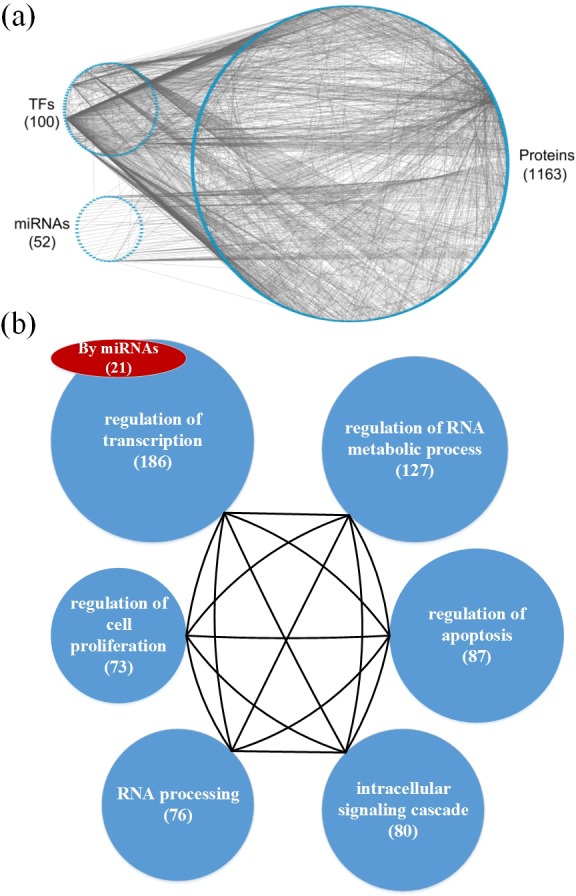
**a.** The GEN of old men and **b.** the associated functional network **a.** The upper left circle represents the TFs (100), the lower left circle represents the miRNAs (52), and the right circle represents the proteins (1163) for the GEN of old men. Links within the “proteins” circle represent PPIs, links between the “TFs” circle and the “proteins” circle represent regulation of TFs on target genes, and links between the “miRNAs” circle and the “proteins” circle represent regulation of miRNAs on target genes in the GEN of old men. **b.** In the functional network of the GEN of old men, the first six enriched GO terms (biological processes) for are regulation of transcription, regulation of RNA metabolic process, regulation of apoptosis, intracellular signaling cascade, RNA processing, and regulation of cell proliferation. The number in parentheses represents the number of proteins involved in each function.

**Figure 6:**
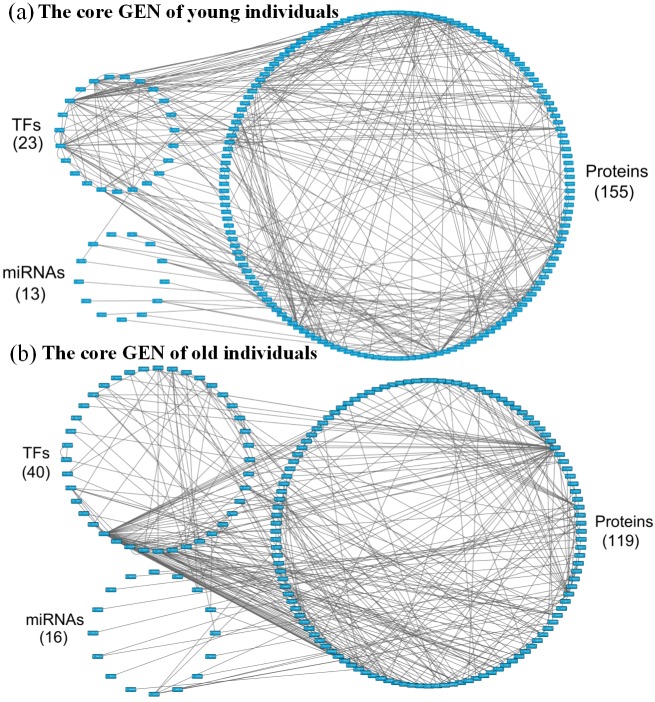
**a.** The core GEN of young individuals from Figure 2a, as determined by the PNP method, and **b.** The core GEN of elderly individuals from Figure 3a, as determined by the PNP method. The upper left circle represents the TFs (23), the lower left circle represents the miRNAs (13), and the right circle represents the proteins (155) for the core GEN of young individuals. **b.** The upper left circle represents the TFs (40), the lower left circle represents the miRNAs (16), and the right circle represents the proteins (119) for the core GEN of elderly individuals. Links within the “proteins” circle represent the significant PPIs, links between the “TFs” circle and the “proteins” circle represent significant regulation of TFs on target genes, and links between the “miRNAs” circle and the “proteins” circle represent significant regulation of miRNAs on target genes in the core GENs of young and elderly individuals.

**Figure 7:**
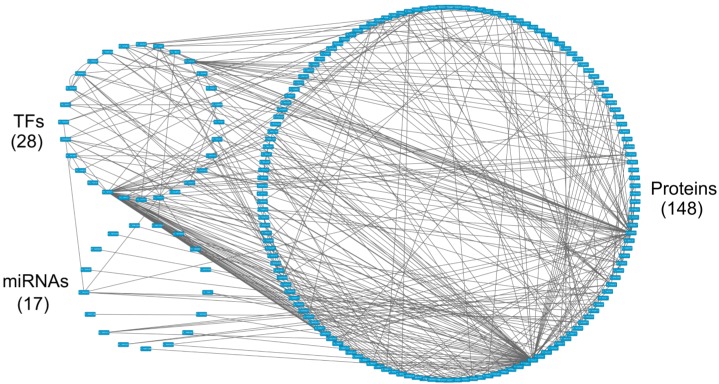
The core GEN of old women from Figure 4a, as determined by the PNP method The upper left circle represents the TFs (28), the lower left circle represents the miRNAs (17), and the right circle represents the proteins (148) for the core GEN of old women. Links within the “proteins” circle represent the significant PPIs, links between the “TFs” circle and the “proteins” circle represent significant regulation of TFs on target genes, and links between the “miRNAs” circle and the “proteins” circle represent significant regulation of miRNAs on target genes in the core GEN of old women.

**Figure 8:**
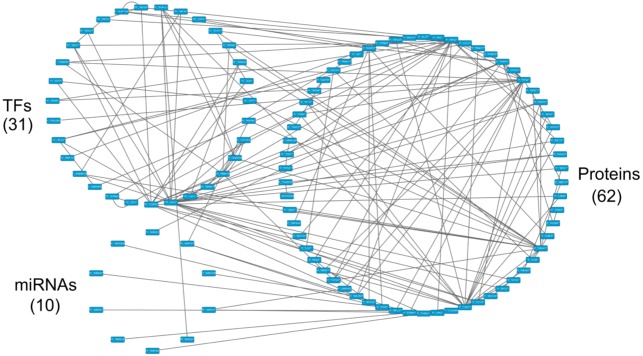
The core GEN of old men from Figure 5a, as determined by the PNP method The upper left circle represents the TFs (31), the lower left circle represents the miRNAs (10), and the right circle represents the proteins (62) for the core GEN of old men. Links within the “proteins” circle represent the significant PPIs, links between the “TFs” circle and the “proteins” circle represent significant regulation of TFs on target genes and links between the “miRNAs” circle and the “proteins” circle represent significant regulation of miRNAs on target genes in the core GEN of old men.

This study is focused on investigating the different molecular mechanisms between young and elderly individuals as well as gender specific distinctive molecular mechanisms between old individuals in the human aging process. Figure 1 summarize the investigation procedure, and the details are also shown in the Materials and Methods section. After identifying the parameters in the PPIN model in (1) and the GRN model in (3) of GENs (see Materials and Methods), the GENs of young and elderly individuals were constructed (Figures 2a and 3a), as well as those for old women and males (Figures 4a and 5a). Using the PNP of GENs, we obtained four core GENs for young and old women and men, as shown in Figures 6a, 6b, 7, and 8, respectively. By intersecting and distinguishing these core proteins, TFs, target genes and miRNAs between young and old core GENs, we identified one common core GEN and two specific core GENs for young and old individuals. Similarly, we also acquired one common core GEN and two specific core GENs for old women and males. In this section, we further discuss the molecular mechanisms of these specific core GENs.

### Functional network and pathway analysis of proteins in the GENs for young and old men and women

Based on the GEN of young and elderly individuals (Figures 2a and 3a), we observed that the number of nodes and links in the elderly is higher than in the young. This illustrates that the GEN of the elderly may contain more pathways. In order to overcome genetic mutations in the aging process, more genetic and epigenetic regulatory mechanisms and pathways are developed to maintain normal cellular functions. This could account for why the GEN of the elderly is more complex than that of the young [18, 28]. Similarly, the GEN of old women contained more nodes and links (Figure 4a) than that of old men (Figure 5a), illustrating that the GEN of old women may contain more pathways. The GEN of old women is developed to eliminate the dysfunction of accumulated genetic mutations in order to maintain normal cellular functions during the aging process, which reflects the increased longevity of old women compared with old men.

We investigated the cellular enrichment functions of these proteins in the GENs of young and old men and women. Using the website tool DAVID https://david.ncifcrf.gov/ and gene ontology analysis, we identified the functional networks of these GENs (Figures 2b, 3b, 4b and 5b) to illustrate the important functional interactions of cellular mechanisms in young and old men and women, respectively. As shown in Figure 2b, the first six enriched GO terms (biological processes) for GENs of young individuals are regulation of transcription, regulation of RNA metabolic process, regulation of apoptosis, intracellular signaling cascade, regulation of transcription from RNA polymerase II promoter, and regulation of cell proliferation. The first six enriched GO terms for GENs of elderly individuals are regulation of transcription, regulation of RNA metabolic process, regulation of apoptosis, intracellular signaling cascade, RNA processing and regulation of cell proliferation (Figure 3b). Although the first six important functions are similar between the cohorts, the number of proteins involved in each function is higher in the elderly than in the young. Furthermore, there are more regulatory miRNAs in the GEN of elderly than in young individuals.

The analysis also demonstrated that the first six enriched GO terms for GENs of old women and men are also similar (Figures 4b and 5b). They include regulation of transcription, regulation of RNA metabolic process, regulation of apoptosis, intracellular signaling cascade, RNA processing and regulation of cell proliferation. Although they share the same biological processes, the number of proteins involved in each function is higher in old women than in old men, and there are more regulatory miRNAs in old women than in old men. In addition, it has been suggested that the human mutation rate is much higher in men than in women, and this increases with paternal age [29, 30]. Accumulation of a large number of abnormal or mutated cells will lead to cancer, which is an aging disease. In the GEN of old women, there is more complexity, which is developed to reduce the dysfunction of accumulated genetic mutations in the aging process. This is reflected in the increased longevity of old women.

The bioinformatics database DAVID, which automatically outputs the results from Kyoto Encyclopedia of Genes and Genomes (KEGG) pathway analysis, was used for the pathway analysis of these proteins. The results of the pathway analysis on the GEN of the elderly are summarized in Table 1. The first eight enriched KEGG pathways are cancer, neurotrophin signaling, MAPK signaling, NOD-like receptor signaling, cytosolic DNA-sensing, B cell receptor signaling, ErbB signaling, and Toll-like receptor signaling pathways. It is possible that the accumulated genetic mutations led to dysregulation of these pathways.

**Table 1:** Pathway analysis of proteins in the GEN of elderly individuals

Pathway analysis		
KEGG pathway	Numbers	*p*-value
**Pathways in cancer**	42	1.7E-4
**Neurotrophin signaling pathway**	19	2.4E-3
**MAPK signaling pathway**	32	3.5E-3
**NOD-like receptor signaling pathway**	12	3.5E-3
**Cytosolic DNA-sensing pathway**	10	1.4E-2
**B cell receptor signaling pathway**	12	1.5E-2
**ErbB signaling pathway**	13	1.8E-2
**Toll-like receptor signaling pathway**	14	2.4E-2

In the GEN of elderly individuals, the first eight enriched KEGG pathways are pathways in cancer, the Neurotrophin signaling pathway, MAPK signaling pathway, NOD-like receptor signaling pathway, Cytosolic DNA-sensing pathway, B cell receptor signaling pathway, ErbB signaling pathway, and Toll-like receptor signaling pathway.

### Pathway analysis of core proteins and the impact of miRNAs and DNA methylation on the elder specific core GEN

For the PNP process, a threshold value is necessary in order to obtain core proteins, TFs, and their target genes, as well as core miRNAs of the core GEN. In the GEN of young individuals, the threshold value *th1* for proteins and TFs in PPI was 4.0, the threshold value *th2* for their target genes in GRN was 0.015, and the threshold value *th3* for miRNAs in epigenetic regulation was 0.01 (Figure S1). In the GEN of the elderly, the threshold value *th1* for proteins and TFs in PPI was 0.6, the threshold value *th2* for their target genes in GRN was 0.35 and the threshold value *th3* for miRNAs in epigenetic regulation was 0.03 (Figure S2).

**Figure 9:**
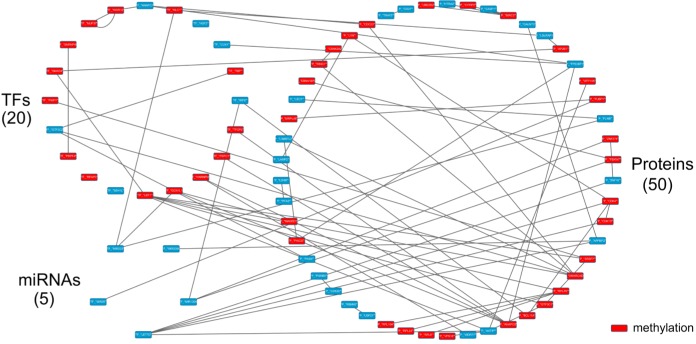
The specific core GEN of elderly individuals, as determined by the distinction between the core GEN of the young and elderly The upper left circle represents the TFs (20), the lower left circle represents the miRNAs (5), and the right circle represents the proteins (50) for the specific core GEN of elderly individuals. The red nodes represent DNA methylation of genes. Links within the “proteins” circle represent the significant PPIs, links between the “TFs” circle and the “proteins” circle represent significant regulation of TFs on target genes, and links between the “miRNAs” circle and the “proteins” circle represent significant regulation of miRNAs on target genes in the specific core GEN of elderly individuals.

The PNP based on the assigned thresholds was used to generate the core GENs of young and old individuals (Figure 6a and 6b). By intersecting and distinguishing these core proteins, TFs and target genes, and miRNAs between the young and core GENs, we identified the common core GEN between the young and elderly, as well as the young and elderly specific core GENs (Figure 9). Several genes demonstrated differences in basal levels (i.e. *k_i_* in (3) are different) between young and elderly individuals. This is mainly due to DNA methylation of corresponding genes, which could potentially be developed to eliminate accumulated genetic mutations and maintain normal cellular functions.

In order to investigate the mechanisms that cause aging, we analyzed the elderly specific core GEN. The bioinformatics database, DAVID, was used to examine the function and related pathways of these core specific proteins (Table 2). The first six enriched KEGG pathways are the Toll-like receptor signaling, T cell receptor signaling, Fc epsilon RI signaling, Neurotrophin signaling, Wnt signaling, and MAPK signaling pathways. We considered that these six pathways might play important roles in the aging process.

**Table 2:** Pathway analysis of core proteins in the specific core GEN of elderly individuals during the aging process

Pathway analysis		
KEGG pathways	Numbers	*p*-value
**Toll-like receptor signaling pathway**	5	9.1E-2
**T cell receptor signaling pathway**	5	1.1E-1
**Fc epsilon RI signaling pathway**	3	2.0E-1
**Neurotrophin signaling pathway**	4	3.8E-1
**Wnt signaling pathway**	4	4.8E-1
**MAPK signaling pathway**	5	5.5E-1

In the specific core GEN of elderly individuals, the first six enriched KEGG pathways are the Toll-like receptor signaling pathway, T cell receptor signaling pathway, Fc epsilon RI signaling pathway, Neurotrophin signaling pathway, Wnt signaling pathway, and MAPK signaling pathway.

In addition, from the identified specific core GEN of elderly individuals (Figure 9), we observed that there are several core genes that are inhibited by core miRNAs. CDK4, APPBP2, ZNF10, CDK13, and AKTIP are inhibited by let-7d, APPBP2 is inhibited by mir-200a, KLC1, FLNB, MYBL1, and GCN1L1 are inhibited by mir-223, FBXW7, ZNF274, and IRF8 are inhibited by mir-130a, and FUBP1 is inhibited by mir-25. Next, we therefore investigated the mechanism by which these core protein-coding genes are regulated by core miRNAs and the related pathways (Table 3). We observed that CDK4 is involved in the p53 signaling, PI3K-Akt signaling, and T cell receptor signaling pathways, FLNB is involved in the MAPK signaling pathway, and ZNF274 is involved in Neurotrophin signaling pathway.

**Table 3:** Core protein-coding genes regulated by core miRNAs in the specific core GEN of elderly individuals and the related pathways

MiRNAs	Genes	Pathways
**Let-7d**	CDK4	p53 signaling pathway PI3K-Akt signaling pathway T cell receptor signaling pathway
**Mir-223**	FLNB	MAPK signaling pathway
**Mir-130a**	ZNF274	Neurotrophin signaling pathway

In the specific core GEN of elderly individuals, CDK4 is inhibited by let-7d, FLNB is inhibited by mir-223, and ZNF274 is inhibited by mir-130a. Moreover, CDK4 is involved in the p53 signaling pathway, PI3K-Akt signaling pathway, and T cell receptor signaling pathway. FLNB is involved in the MAPK signaling pathway, and ZNF274 is involved in Neurotrophin signaling pathway.

**Figure 10:**
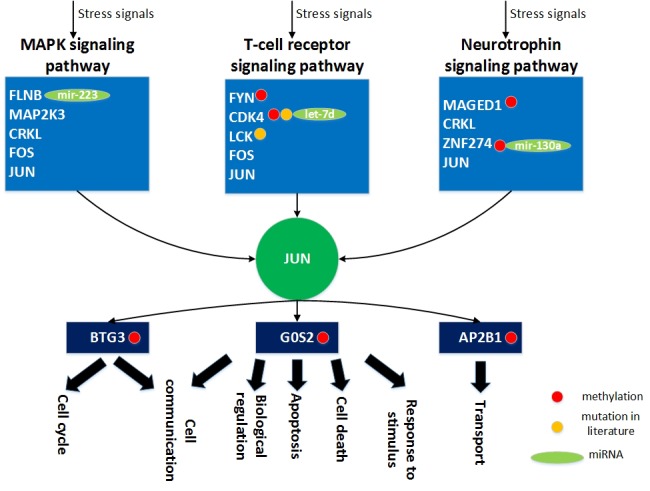
The role of miRNA regulation and DNA methylation on the human aging mechanism based on the specific core GEN of the elderly During the aging process, FLNB, MAP2K3, CRKL, FOS and JUN play key roles in the MAPK signaling pathway. In addition, FYN, CDK4, LCK, FOS, and JUN are involved in T-cell receptor signaling pathway, and MAGED1, CRKL, ZNF274 and JUN are involved in neurotrophin signaling pathway. In order to overcome the cellular dysfunction caused by accumulated genetic mutations during the aging process, the transcription factor JUN can be activated by a wide variety of stress signals, including the MAPK signaling, T-cell receptor signaling, and neurotrophin signaling pathways. This results in various cellular responses, including cell cycle, cell communication, apoptosis, cell death, biological regulation, cell transport, and response to stimulus. In this figure, the light blue blocks represent the proteins involved in the indicated pathways, the dark green node represents the TF the dark blue blocks represent the TFs target genes, the light green nodes represent the miRNAs, the red nodes represent DNA methylation of genes, the yellow nodes represent the mutation of genes, arrows represent activation of proteins, and link represents interaction between these two proteins.

The results of the pathway analysis demonstrate the importance of these core miRNAs in the elderly specific core GEN, as a significant number of mutations in these pathways often lead to cellular dysfunction. We observed that there are three core miRNAs, mir-223, let-7d and mir-130a, that regulate FLNB, CDK4, and ZNF274, respectively (Figure 10). Those genes are involved in the MAPK signaling, T cell receptor signaling, and Neurotrophin signaling pathways, respectively. Because these three miRNAs and genes only appear in the elderly specific core GEN, we speculate that they play critical roles in the aging process.

### Pathway analysis of core proteins and the impact of miRNAs and DNA methylation on the specific core GEN of old women

As previously mentioned, in the PNP process it is necessary to select threshold values in order to obtain core proteins, TFs and their target genes, and core miRNAs of the core GEN. In the GEN of old women, the threshold value *th1* for proteins and TFs in PPI was 0.5, the threshold value *th2* for their target genes in GRN was 0.4, and the threshold value *th3* for miRNAs in epigenetic regulation was 0.1 (Figure S3). In the GEN of old men, the threshold value *th1* for proteins and TFs in PPI was 0.8, the threshold value *th2* for their target genes in GRN was 0.003, and the threshold value *th3* for miRNAs in epigenetic regulation was 0.003 (Figure S4).

**Figure 11:**
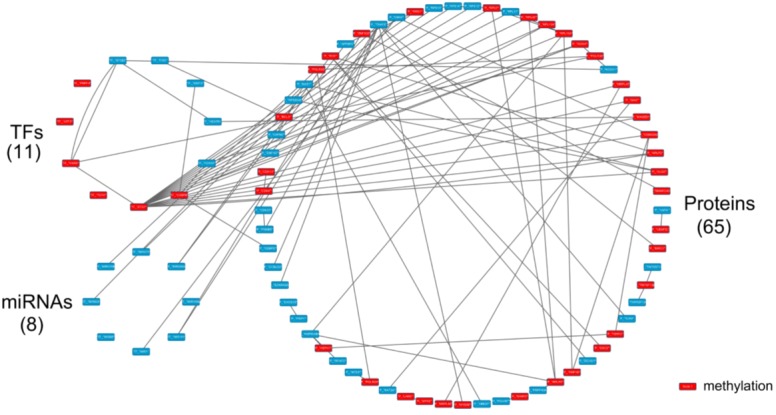
The specific core GEN of old women as determined by the distinction between the core GEN of old women and men The upper left circle represents the TFs (11), the lower left circle represents the miRNAs (8) and the right circle represents the proteins (65) for the specific core GEN of old women. The red nodes represent DNA methylation of genes. Links within the “proteins” circle represent the significant PPIs, links between the “TFs” circle and the “proteins” circle represent significant regulation of TFs on target genes and links between the “miRNAs” circle and the “proteins” circle represent significant regulation of miRNAs on target genes in the specific core GEN of old women.

**Figure 12:**
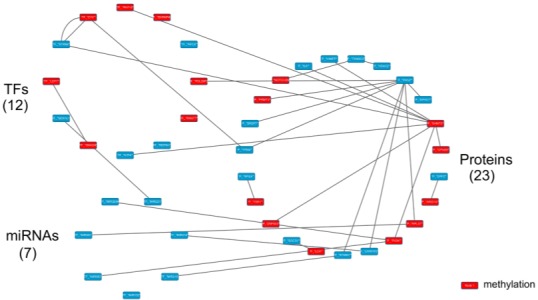
The specific core GEN of old men as determined by the distinction between the core GEN of old women and old men The upper left circle represents the TFs (12), the lower left circle represents the miRNAs (7), and the right circle represents the proteins (23) for the specific core GEN of old men. The red nodes represent DNA methylation of genes. Links within the “proteins” circle represent the significant PPIs, links between the “TFs” circle and the “proteins” circle represent significant regulation of TFs on target genes and links between the “miRNAs” circle and the “proteins” circle represent significant regulation of miRNAs on target genes in the specific core GEN of old men.

Using the PNP method based on thresholds in Figure S3 and Figure S4, we obtained the old female and male core GENs (Figures 7 and 8). By intersecting and distinguishing these core proteins, TFs and target genes, and miRNAs between old female and male core GENs, we identified the common core GEN between old women and males, the old female specific core GEN (Figure 11), and the old male specific core GEN (Figure 12). We observed that there were greater differences in the basal levels of genes (i.e. *k_i_* in (3) are different) in the specific core GEN of old women than in that of old men, which are mainly due to DNA methylation of corresponding genes. We speculated that old women probably developed these methylated genes in an effort to eliminate the dysfunction of accumulated genetic mutations in the aging process. We analyzed the old female and male specific core GENs to determine the distinctive mechanisms between old women and males that contribute to the gender-specific differences in the aging process.

The results of the pathway analysis of the specific core GEN of old women are summarized in Table 4. The first four enriched KEGG pathways are the Toll-like receptor, MAPK, NOD-like receptor, and Neurotrophin signaling pathways. This indicates that these pathways are critical to the aging process in women.

**Table 4:** Pathway analysis of core proteins in the specific core GEN of old women during the aging process

Pathway analysis		
KEGG pathways	Numbers	*p*-value
**Toll-like receptor signaling pathway**	5	3.8E-2
**MAPK signaling pathway**	8	4.9E-2
**NOD-like receptor signaling pathway**	3	1.8E-1
**Neurotrophin signaling pathway**	4	2.0E-1

In the specific core GEN of old women, the first four enriched KEGG pathways are the Toll-like receptor signaling pathway, MAPK signaling pathway, NOD-like receptor signaling pathway, and Neurotrophin signaling pathway.

In addition, we found that there are many core genes that are inhibited by the core miRNAs (Figure 11). TRAF6 and TAOK3 are inhibited by mir-373, GYK and APPBP2 are inhibited by mir-200a, TRAF6, TAOK3 and ZNF302 are inhibited by mir-141, CBX2 is inhibited by mir-1, APBB1 is inhibited by mir-148b, POLR3D is inhibited by mir-374b, NAE1 is inhibited by mir-503, and GTF2I is inhibited by mir-98.

Next, we investigated the pathways and mechanisms involved in the regulation of these core protein-coding genes by core miRNAs. The results are summarized in Table 5. TRAF6 is involved in the MAPK signaling, NF-kappa B signaling, Toll-like receptor signaling, NOD-like receptor signaling, RIG-I-like receptor signaling, and Neurotrophin signaling pathways. In addition, TAOK3 is involved in the MAPK signaling pathway, POLR3D is involved in metabolic and cytosolic DNA-sensing pathways, and GTF2I is involved in the cGMP-PKG signaling pathway. Based on these findings, it is possible that the observed negative regulation of miRNAs were developed as a protective mechanism to ensure normal cellular functions.

**Table 5:** Core protein-coding genes regulated by core miRNAs in the specific core GEN of old women and the related pathways

MiRNAs	Genes	Pathways
**Mir-141** **Mir-373**	TRAF6	MAPK signaling pathway NF-kappa B signaling pathway Toll-like receptor signaling pathway NOD-like receptor signaling pathway RIG-I-like receptor signaling pathway Neurotrophin signaling pathway
**Mir-141** **Mir-373**	TAOK3	MAPK signaling pathway
**Mir-374b**	POLR3D	Metabolic pathways Cytosolic DNA-sensing pathway
**Mir-98**	GTF2I	cGMP-PKG signaling pathway

In the specific core GEN of old women, TRAF6 and TAOK3 are inhibited by mir-141 and mir-373, POLR3D is inhibited by mir-374b, and GTF2I is inhibited by mir-98. Moreover, TRAF6 is involved in the MAPK signaling pathway, NF-kappa B signaling pathway, Toll-like receptor signaling pathway, NOD-like receptor signaling pathway, RIG-I-like receptor signaling pathway, and Neurotrophin signaling pathway. TAOK3 is involved in the MAPK signaling pathway, POLR3D is involved in metabolic pathways and the cytosolic DNA-sensing pathway, and GTF2I is involved in the cGMP-PKG signaling pathway.

**Figure 13:**
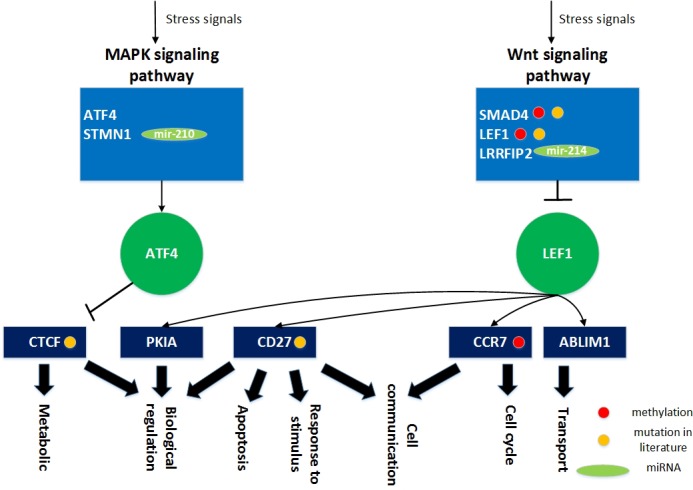
The role of miRNA regulation and DNA methylation in gender specific aging mechanisms based on the specific core GEN of old men During the aging process, ATF4 and STMN1 are involved in the MAPK signaling pathway, and SMAD4, LEF1, and LRRFIP2 play key roles in the Wnt signaling pathway. Since these genetic and epigenetic regulation observed cannot overcome the dysfunction due to accumulated genetic mutations during the aging process, the transcription factor ATF4 can be activated and LEF1 can be inhibited by a wide variety of stress signals, including the MAPK and Wnt signaling pathways. This results in deregulation of various cellular responses, including cell cycle, apoptosis, metabolism, cell communication, cell transport, biological regulation, and response to stimulus. In this figure, the light blue blocks represent the proteins involved in the indicated pathways, the deep green nodes represent the TFs, the deep blue blocks represent the TFs target genes, the light green nodes represent the miRNAs, the red nodes represent DNA methylation of genes, the yellow nodes represent the mutation of genes, arrows represent activation of proteins, and the link represents interaction between these two proteins.

Pathway analyses demonstrated that there are two core miRNAs, mir-373 and mir-141 (Figure 13). TAOK3 and TRAF6 are inhibited by mir-373, and are involved in the MAPK signaling pathway. However, TRAF6 is also inhibited by mir-141, and is also involved in the Toll-like receptor signaling pathway. Because these two miRNAs and genes only appear in the old female specific core GEN it is likely that these two miRNAs, genes, and pathways play a critical role in the female-specific aging process.

### Pathway analysis of core proteins and the impact of miRNAs and DNA methylation on the specific core GEN of old men

Using the bioinformatics database, DAVID, we investigated the molecular pathways related to the core proteins in the old male specific core GEN (Table 6). It is noteworthy that only one enriched KEGG pathway is related to cancer.

**Table 6:** Pathway analysis of core proteins in the specific core GEN of old men during the aging process

Pathway analysis		
KEGG pathways	Numbers	*p*-value
Pathway in cancer	3	3.0E-1

In the specific core GEN of old men, only one KEGG pathway is enriched in the aging process.

In addition, from our specific core GEN of old men (Figure 12), we observed that there are many core genes that are inhibited by the core miRNA. For instance, TUBG1 is inhibited by mir-152, STMN1 is inhibited by mir-210, LRRFIP2 and UNG are inhibited by mir-214, GCN1L1 is inhibited by mir-221, RPL37 is inhibited by mir-381, and PIGN is inhibited by mir-320a and mir-653. In Table 7, we show the specific pathways that these core protein-coding genes are involved in. STMN1 is involved in the MAPK signaling pathway, and LRRFIP2 is involved in the Wnt signaling pathway.

**Table 7:** Core protein-coding genes regulated by core miRNAs in the specific core GEN of old men and the related pathways

MiRNAs	Genes	Pathways
**Mir-210**	STMN1	MAPK signaling pathway
**Mir-214**	LRRFIP2	Wnt signaling pathway

In the specific core GEN of old men, STMN1 is inhibited by mir-210, and LRRFIP2 is inhibited by mir-214. In addition, STMN1 is involved in the MAPK signaling pathway, and LRRFIP2 is involved in the Wnt signaling pathway.

The results of the pathway analysis indicate no intersection between the two findings (Figure 13). Therefore, we can only investigate how these two core protein-coding genes are regulated by core miRNAs in the old male specific core GEN and the associated pathways. We observed that there are two core miRNAs, mir-210 and mir-214, that regulate STMN1 and LRRFIP2, respectively which are involved in the MAPK and Wnt signaling pathways, respectively. These two miRNAs and genes only appear in the old male specific core GEN indicating that they are important for the male-specific aging process.

## DISCUSSION

### The role of miRNA regulation and DNA methylation in human aging based on the specific core GEN of elderly individuals

Aging is a complex process driven by diverse molecular pathways. Based on analysis of the specific core GEN of elderly individuals, we demonstrate that FLNB, MAP2K3, CRKL, FOS and JUN are involved in the MAPK signaling pathway (Figure 10). This pathway is activated by diverse extracellular and intracellular stimuli including peptide growth factors, cytokines, hormones, and various cellular stressors such as oxidative and endoplasmic reticulum stress. It has been suggested that the MAPK pathway regulates a variety of cellular activities including proliferation, differentiation, survival, and death. Dysregulation of the MAPK signaling pathway has been implicated in the development of many human diseases and aging [31].

In addition, we found that FYN, CDK4, LCK, FOS, and JUN are involved in the T-cell receptor signaling pathway. This signaling pathway, in response to antigen recognition, plays a central role in the adaptive immune response [32]. T-cell activation by foreign antigens induces antigen specific T-cell clonal expansion and differentiation, and this response is regulated by signal transduction pathways initiated by antigen receptors and costimulatory molecules [33].

Additionally, MAGED1, CRKL, ZNF274, and JUN are involved in the neurotrophin signaling pathway. This pathway is activated by neurotrophins binding to cognate receptors, which have been shown to regulate almost all aspects of neuronal development and function, including precursor proliferation and commitment, cell survival, axon and dendrite growth, membrane trafficking, synapse formation and function, as well as glial differentiation and interactions with neurons [34].

Humans possess the capability to respond to a variety of external signals through cell surface receptors. In Figure 10, extracellular and intracellular stimuli through the MAPK signaling, T-cell receptor signaling, and neurotrophin signaling pathways lead to activation of JUN, and result in various cellular responses through transcriptional regulation of its target genes. The transcription factor JUN can activate many target genes, including BTG3 (*b_ij_* = 0.150252), G0S2 (*b_ij_* = 0.246038) and AP2B1 (*b_ij_* = 0.043467). BTG3 is involved in cell cycle and cell communication, G0S2 is involved in apoptosis, cell communication, cell death, biological regulation and response to stimuli, and AP2B1 is involved in cell transport.

We speculate that increased genetic and epigenetic regulatory mechanisms and pathways are developed during the aging process in order to maintain normal cellular function and overcome accumulated genetic mutations. Accumulation of a large number of abnormal or mutated cells is part of the aging process. Mutation of CDK4 was found to be associated with a variety of cancers [35] and mutation of LCK was found to be associated with immunodeficiency [36]. We observed that expression of these two genes vary significantly between young and old individuals (*p* < 0.05), which supports the above findings.

In addition, we found seven specific genes that demonstrate differing basal expression levels (i.e. *k_i_* in (3) are different) between young and elderly individuals, mainly due to DNA methylation. They are FYN, CDK4, MAGED1, ZNF274, BTG3, G0S2, and AP2B1. Analysis of the MethHC database (a database of DNA methylation and gene expression in Human Cancer http://methhc.mbc.nctu.edu.tw/php/index.php) [37] demonstrated that DNA methylation of FYN, CDK4, MAGED1, ZNF274, BTG3, G0S2 and AP2B1 varies significantly (*p* < 0.05) between tumor and normal samples, which support our findings since cancer is an aging disease. In addition, FLNB is inhibited by mir-223 (*c_il_* = −0.21954), CDK4 is inhibited by miRNA let-7d (*c_il_* = −0.50305), and ZNF274 is inhibited by mir-130a (*c_il_* = −0.10597). These results were observed only in the specific core GEN of elderly individuals. The differences between the young and elderly specific core GENs might be responsible for human aging mechanisms. In addition, it has been reported that FOS and JUN are aging-related human genes (Figure 10 and GenAge: The Ageing Gene Database http://genomics.senescence.info/genes/) [38], which also support our results.

In order to overcome the accumulated mutation of genes in the aging process, the transcription factor JUN can be activated by a wide variety of stress signals in the aging process, including the MAPK signaling, T-cell receptor signaling, and neurotrophin signaling pathways. This results in various cellular responses, including cell cycle, cell communication, apoptosis, cell death, biological regulation, cell transport and response to stimulus. If not, aging is accompanied by a decline in the function of the immune system, which increases susceptibility to infections. In addition, dysfunction of the cell cycle results in a decreased ability of cells to arrest at key checkpoints and also leads to the survival of abnormal or mutated cells that would normally die, resulting in tumorigenesis. Accumulation of a large number of abnormal or mutated cells leads to aging. Furthermore, a defective apoptotic processes results in a wide variety of diseases, such as cancer. In short, aging is characterized by deterioration in the maintenance of homeostatic processes over time [18], leading to functional decline and increased risk for disease and death. Therefore, drugs designed to target these DNA methylated genes (BTG3, G0S2 and AP2B1) or genes inhibited by miRNAs, mir-223, let-7d, and mir-130a (FLNB, CDK4 and ZNF274) may delay aging. Therefore, according to the databases including GeneCards database and ZINC database, a multiple drug combination comprising phenylalanine, cholesterol, and palbociclib was designed for delaying the aging process (Figure S5).

### The role of miRNA regulation and DNA methylation on human gender aging mechanisms based on the specific core GEN of old women

**Figure 14:**
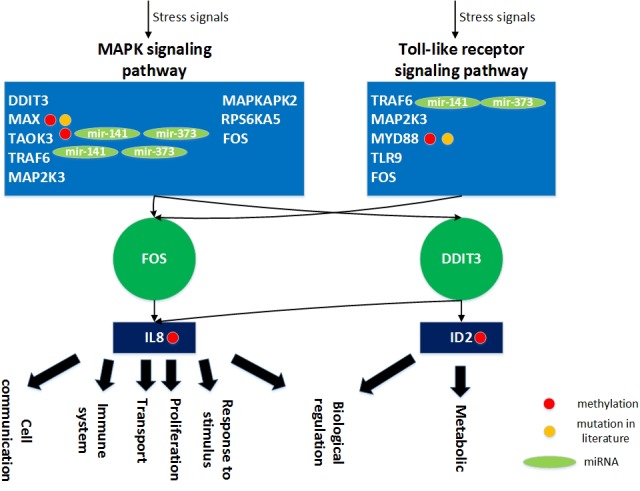
The role of miRNA regulation and DNA methylation on gender specific aging mechanisms based on the specific core GEN of old women In the aging process, DDIT3, MAX, TAOK3, TRAF6, MAP2K3, MAPKAPK2, RPS6KA5, and FOS are involved in the MAPK signaling pathway and TRAF6, MAP2K3, MYD88, TLR9, and FOS are involved in the Toll-like receptor signaling pathway. In order to overcome the dysfunction caused by accumulated genetic mutations during the aging process, the transcription factor FOS and DDIT3 can be activated by a wide variety of stress signals, including the MAPK signaling and Toll-like receptor signaling pathways. This results in various cellular responses, including immune system activation, cell proliferation, metabolism, cell communication, cell transport, biological regulation, and response to stimulus. In this figure, the light blue blocks represent the proteins involved in the indicated pathways, the deep green nodes represent the TFs, the deep blue blocks represent the TFs target genes, the light green nodes represent the miRNAs, the red nodes represent DNA methylation of genes, the yellow nodes represent the mutation of genes, arrows represent activation of proteins, and the link represents interaction between these two proteins.

Analysis of gender specific aging mechanisms based on the specific core GEN of old women demonstrated that DDIT3, MAX, TAOK3, TRAF6, MAP2K3, MAPKAPK2, RPS6KA5, and FOS are involved in the MAPK signaling pathway (Figure 14). The MAPK family plays an important role in complex cellular programs such as proliferation, differentiation, development, transformation, and apoptosis [39].

In addition, TRAF6, MAP2K3, MYD88, TLR9, and FOS are involved in the Toll-like receptor signaling pathway. Toll-like receptors (TLRs) play an essential role in the activation of innate immunity by recognizing specific patterns of microbial components [40]. Stimulation of different TLRs induces distinct patterns of gene expression, which not only lead to activation of innate immunity but also instruct the development of antigen-specific acquired immunity [41].

Extracellular and intracellular stimuli through the MAPK signaling and Toll-like receptor signaling pathways lead to activation of FOS and DDIT3, and result in various cellular responses through transcriptional regulation of its target genes (Figure 14). The transcription factor FOS can activate the IL8 gene (*b_ij_* = 1.136866). IL8 is involved in a number of cellular functions of the immune system, cell proliferation, cell communication, cell transport, biological regulation, and the response to various stimuli. In addition, the transcription factor DDIT3 can activate many target genes, including IL8 (*b_ij_* = 1.143526) and ID2 (*b_ij_* = 0.458447). The functions of ID2 include metabolism and biological regulations.

Accumulation of mutations play a key role in the aging process, and it was shown that mutation of MAX is associated with hereditary pheochromocytoma [42], whereas mutation of MYD88 was found to be associated with many hematological malignancies [43]. We found that the expression of these two genes vary significantly between young and old individuals (*p* < 0.05), which supports the above results.

In addition, we found that five specific genes, MAX, TAOK3, MYD88, IL8 and ID2, have varying basal expression levels (i.e. *k_i_* in (3) are different) between young and old women, which is mainly due to the DNA methylation of corresponding genes. It has been reported that DNA methylation and the expression levels of these five genes vary significantly between tumor and normal samples (*p* < 0.05; *via* MethHC) [37], supports our results. In addition, TAOK3 and TRAF6 are inhibited by mir-141 (*c_il_* = −0.2235-TAOK3; *c_il_* = −0.36144-TRAF6) and mir-373 (*c_il_* = −0.10454-TAOK3; *c_il_* = −0.12645-TRAF6). Mutations in these genes, inhibition of miRNAs, and DNA methylation only appear in the specific core GEN of old women. In addition, it has been reported that DDIT3, MAX, and FOS are aging-related human genes (Figure 14; GenAge database). Other aging-related genes were not identified in our analyses.

The transcription factors FOS and DDIT3 can be activated by a wide variety of stress signals in the female aging process, including the MAPK signaling and Toll-like receptor signaling pathways, resulting in various cellular responses, including immune activation, cell proliferation, metabolism, cell communication, cell transport, biological regulation, and response to various stimuli. If these transcription factors are not activated during the aging process, the innate immune system becomes dysregulated, which is characterized by persistent inflammatory responses that involve multiple immune and non-immune cell types. This leads to proliferation of abnormal or mutated cells and the increased incidence of tumorigenesis. Many tumors accumulate in the body during aging. In addition, many metabolic pathways decline with age, and this is a major consequence of the aging process, which induces susceptibility to a number of diseases. Therefore, drugs designed to target IL8, ID2, TRAF6, or TAOK3 may improve the aging process in women. Finally, a multiple drug combination comprising adenosine triphosphate, sunitinib, bromodeoxyuridine, and rivanicline was designed for delaying the aging process in women based on GeneCards and ZINC databases (Figure S5).

### The role of miRNA regulation and DNA methylation in the human aging mechanism based on the specific core GEN of old men

Analysis of the specific core GEN of old men revealed that ATF4 and STMN1 are involved in the MAPK signaling pathway (Figure 13). The MAPK signal pathway regulates a wide variety of cellular functions in response to cellular stresses [53]. In addition, SMAD4, LEF1, and LRRFIP2 are involved in the Wnt signaling pathway. It has been suggested that the Wnt signaling pathway has important functions in stem cell biology, cardiac development and differentiation, angiogenesis, cardiac hypertrophy, cardiac failure, and aging [45]. Aberrant Wnt signaling underlies a wide range of pathologies in humans [46].

We show that extracellular and intracellular stimuli through the MAPK signaling and Wnt signaling pathways lead to activation of ATF4 and inhibition of LEF1, resulting in various cellular responses through transcriptional regulation of target genes (Figure 13). The transcription factor ATF4 can inhibit the CTCF gene (*b_ij_*= −0.57079), which plays critical roles in metabolism and biological regulation. In addition, the transcription factor LEF1 can activate many target genes, including ABLIM1 (*b_ij_*= 0.659135), CCR7 (*b_ij_* = 0.833707), CD27 (*b_ij_*= 0.850484), and PKIA (*b_ij_* = 0.485246). ABLIM1 is important for cell transport, CCR7 is involved in cell cycle and cell communication, CD27 plays a role in apoptosis, cell communication, biological regulation, and response to stimulus, and PKIA is necessary for biological regulation.

As previously mentioned, accumulation of genetic mutations is highly correlated with the aging process, and it has been shown that mutation of SMAD4 is associated with pancreatic cancer [47], juvenile polyposis syndrome [48], and hereditary hemorrhagic telangiectasia syndrome [49]. Moreover, mutation of LEF1 was found to be associated with somatic sebaceous tumors [50], whereas mutation of CTCF is associated with invasive breast cancers [51], prostate cancers [52], and Wilms' tumors [52]. Mutation of CD27 is associated with lymphoproliferative syndrome 2 [53]. Interestingly, the expression levels of these four genes differ significantly (*p* < 0.05) between young and old men in our data, which supports the above findings.

In addition, we found that three specific genes have differing basal level expression (i.e. *k_i_* in (3) are different) between young and old men, which is mainly due to DNA methylation of corresponding genes. These genes are SMAD4, LEF1, and CCR7. It has been reported that DNA methylation of SMAD4, LEF1, and CCR7 demonstrate varying expression levels (*p* < 0.05) between tumor and normal samples (MethHC database) [37], which support our results. Additionally, STMN1 is inhibited by mir-210 (*c_il_* = −0.25071) and LRRFIP2 is inhibited by the mir-214 (*c_il_* = −0.48908). These genetic mutations, miRNA inhibitions, and DNA methylation of genes only appear in the old male specific core GEN. These differences might account for the aging mechanisms in old men. It has been suggested that the mutation rate is much higher in men than in women, and increases with age. The specific core GEN of old men is less developed to eliminate dysfunctions due to accumulated genetic mutations in the aging process compared to old women, which reflects the shorter life span of old men. It has been reported that CD27 is an aging-related human gene (GenAge).

Since these genetic and epigenetic regulations cannot overcome the accumulated genetic mutations in the aging process, the transcription factors ATF4 can be activated and LEF1 can be inhibited by a wide variety of stress signals in the male-specific aging process. This includes the MAPK signaling and Wnt signaling pathways, which result in dysregulation of various cellular responses, including cell cycle, apoptosis, metabolism, cell communication, cell transport, biological regulation, and the response to various stimuli. Furthermore, apoptosis enables an organism to eliminate unwanted and defective cells through an orderly process of cellular disintegration. Improper regulation of apoptosis contributes to disorders such as cancer, viral infection, and autoimmune diseases. Moreover, the cell cycle represents a series of events responsible for cell duplication. Dysregulation of the cell cycle leads cancer development. Medical statistics indicate that men are more likely than women to get cancer. This may explain why men have a lower average life expectancy compared to women. Therefore, designing drugs to the DNA methylated genes, CTCF, CD27 and CCR7, or the genes inhibited by miRNAs, mir-210 and mir-214, STMN1 and LRRFIP2, may improve the male-specific aging process. Furthermore, a multiple drug combination comprising vinblastine, paclitaxel, and map4 was designed for delaying the male-specific aging process based on GeneCards and ZINC databases (Figure S5).

### The overall mechanism of human aging

Aging is characterized by a general decline in cellular function, which ultimately affects homeostasis of the entire body [18]. Aging is an inevitable part of life and comes with all sorts of physical and mental ailments, including common metabolism, inflammation, immune decline, and cancer [54]. Extracellular and intracellular activation of various pathways, such as the MAPK signaling, T-cell receptor signaling, neurotrophin signaling, Toll-like receptor signaling, and Wnt signaling pathways lead to various cellular responses. Genetic mutations lead to dysregulation of pathways and dysfunction of various cellular responses necessary during the aging process. In order to overcome these dysfunctions, genetic and epigenetic regulation is enhanced to maintain normal cellular functions. In addition, it has been suggested that the human mutation rate is much higher in men than in women and increases with paternal age. Accumulation of a large number of abnormal or mutated cells will lead to cancer, which is an aging disease. For old women, GEN is more developed than that of old men, which reflects the increased longevity of old women.

## CONCLUSIONS

In this study, to investigate human aging mechanisms from peripheral blood mononuclear cells, the GENs of young and old men and women were constructed based on their corresponding microarray data, miRNA data, methylation data, and database mining *via* the least squares parameter estimation method, AIC order detection model, and the Student's *t*-test. The core GENs were obtained using the PNP method based on PCA. However, the common core and specific core GENs were acquired using the intersection and distinction of core proteins, TFs, target genes, and miRNAs in core GENs between the young and elderly, and between old women and males. We found that in the specific core GEN of elderly individuals, FLNB, CDK4, and ZNF274 are inhibited by mir-223, let-7d, and mir-130a, respectively, and DNA methylation of FYN, CDK4, MAGED1 and ZNF274 in order to overcome dysregulation of the MAPK signaling, T-cell receptor signaling, and neurotrophin signaling pathways, as well as deregulated cell cycle and apoptosis processes. The specific core GEN of old women demonstrated that TAOK3 and TRAF6 are inhibited by mir-141 and mir-373, respectively, and DNA methylation of MAX, TAOK3, and MYD88 in order to overcome dysregulation of the MAPK signaling and Toll-like receptor signaling pathways as well as dysfunctions of the immune system, proliferation, and metabolism. The specific core GEN of old men showed that STMN1 and LRRFIP2 are inhibited by mir-210 and mir-214, respectively, and DNA methylation of SMAD4 and LEF1, which may lead to dysregulation of the MAPK signaling and Wnt signaling pathways as well as deregulation of the cell cycle and apoptosis, thus resulting in cancer. The results obtained from the present study provides a platform for useful medical therapy to facilitate the development of new anti-aging drugs.

## MATERIALS AND METHODS

### Overview of the construction of common and specific core GENs of human aging

Figure 1 shows an outline of the process for constructing the common and specific core GENs of human aging. Microarray data of young and old samples, miRNA data from peripheral blood mononuclear cells, as well as the miRNA, methylation, TF-gene, and BioGRID databases were searched in order to construct candidate GENs. These GENs consisted of interactive candidate GRN, PPI, and miRNA regulation networks. In order to avoid false positives, we applied system modeling and least square estimation [55]. The core GENs for young and old men and women were obtained by PNP. The intersection of young and old core GENs is called a common core GEN, whereas the distinction between young and old core GENs is referred to as the young and/or old specific core GENs. We investigated the gender-specific aging mechanisms in young and old individuals based on their specific core GENs as well as data obtained from analyzing peripheral blood mononuclear cells from the respective individuals.

### Data selection and preprocessing

Microarray datasets were obtained from the NCBI Gene Expression Omnibus (GEO). A study by Marttila *et al.* demonstrated gender-specific changes during aging of the human immune system based on transcriptomic analysis of peripheral blood mononuclear cells derived from elderly individuals and young controls [56]. The use of peripheral blood mononuclear cells may provide a useful tool to study human aging [57, 58]. Using the GEO, we obtained the data from this study for young and elderly individuals (GSE65219) [56]. The sample numbers for the young (19-30 years of age) and elderly (90-99 years of age) groups were 30 and 146, respectively. In addition, 103 and 43 of the 146 samples were old women and males, respectively.

The PPI candidate dataset for Homo sapiens was obtained from the Biological General Repository for Interaction Database (BioGRID, downloaded in March 2015). BioGRID is a freely accessible database of physical and genetic interactions available at http://www.thebiogrid.org [59]. More than 700,000 interactions are available from major model organism species and from more than 50,000 publications.

A TF-gene regulation dataset is available at The Human Transcriptional Regulation Interactions database (HTRIdb) [60] and Integrated Transcription Factor Platform (ITFP) [61]. HTRIdb has been populated with 284 TFs totaling 51871 regulations. In addition, ITFP has been populated with 4015 TFs and 69496 regulations.

The miRNA regulation dataset was collected from starBase v2.0 http://starbase.sysu.edu.cn/ and mirTarBase http://mirtarbase.mbc.nctu.edu.tw/. The miRTarBase database provides the most current and comprehensive information of experimentally validated miRNA-target interactions [62]. All the above large databases were used as candidates for identifying GENs of young and old male and female samples.

### Selection of a protein pool and construction of the candidate GENs for young and old men and women

In order to construct the candidate GENs, which are integrated through gene expression with microarray data for young and elderly samples, miRNA data, and the miRNA, TF-gene, and BioGRID databases were used based on the following two steps to select the protein pool. First, gene expression values were overlaid onto the corresponding proteins. Then, a one-way analysis of variance (ANO VA) was performed to select differentially expressed proteins (*p* < 0.05). The null hypothesis (*H_0_*) assumes that the average protein expression levels of young and elderly samples are the same. Proteins with *p*-values < 0.05 were selected for the protein pool to construct the candidate GENs containing candidate GRN, PPIN, and the miRNA regulatory network. A total of 2306 differentially expressed proteins between young and elderly samples were selected as candidate GENs. Therefore, the candidate network consisted of 4581 PPIs, and 4400 TFs and 53099 miRNAs regulations.

### The regression model of the GENs for young and old men and women

A candidate GEN contains false positive interactions and regulations because miRNA, TF-gene, and BioGRID databases include all experimental conditions and various experimental environments. The false positives of a candidate GEN should therefore be pruned by using real microarray and miRNA data through the system identification and system order detection method. Two regression models, the protein association model and gene regulatory model, are used to characterize the GEN in cells.

The PPIs of the protein *i* in the candidate PPIN can be described by the following protein association model:

$$y_{i}\left(n\right)=\sum_{j=1,i\neq j}^{N_{i}}a_{ij}y_{i}\left(n\right)y_{j}\left(n\right)+h_{i}+v_{i}\left(n\right),\text{ for }i=1,2,\ldots ,N$$ (1)

where *y_i_*(*n*) represents the mRNA expression level of the protein *i* for the sample *n*, *y_j_*(*n*) denotes the mRNA expression level of the *j*^th^ protein interacting with protein *i* for the sample *n*, *a_ij_* represents the association ability between protein *i* and its interactive protein *j*, *N_i_* indicates the number of proteins interacting with protein *i* in the candidate PPIN, *h_i_* is the basal expression level of protein *i*, and *v_i_*(*n*) characterizes the stochastic noise of the *i*^th^ protein for sample *n*. The model demonstrates that protein *i* is affected by *N_i_* proteins, the basal expression level, and stochastic noise. The PPI association equation of protein *i* in (1) can be represented by following regression form:

$$\begin{array}{ll} y_{i}\left(n\right)= & \left[y_{i}\left(n\right)y_{1}\left(n\right)\;\cdots \;y_{i}\left(n\right)y_{j-1}\left(n\right)\;y_{i}\left(n\right)y_{j+1}\left(n\right)\;\cdots \;y_{i}\left(n\right)y_{N_{i}}\left(n\right)\;1\right]\times \\ & \left[\begin{array}{c} a_{i1}\\ \vdots \\ a_{ij-1}\\ a_{ij+1}\\ \vdots \\ a_{{iN}_{i}}\\ h_{i} \end{array}\right]+v_{i}\left(n\right)=\Phi_{i}\left(n\right)\Theta_{i}+v_{i}\left(n\right),\text{ for }i=1,2,\ldots ,N \end{array}$$ (2)

In addition, the TF and miRNA regulation of the target gene *i* can be described using the following gene regulatory model:

$$x_{i}\left(n\right)=\sum_{j=1,i\neq j}^{N_{i}}b_{ij}y_{j}\left(n\right)-\sum_{l=1}^{O_{i}}c_{il}mir_{l}\left(n\right)+k_{i}+w_{i}\left(n\right),\text{ for }i=1,2,\ldots ,N$$ (3)

where *x_i_*(*n*) represents the mRNA expression level of target gene *i* for sample *n*, *y_i_*(*n*) denotes the mRNA expression level of the *j*^th^ TF binding to the target gene *i* for sample *n*, *b_ij_* indicates the regulatory ability of the *j*^th^ TF binding to the *i*^th^ target gene, *mir_i_*(*n*) represents the expression level of the *l*^th^ miRNA interacting with target gene *i* for sample *n*, *c_il_* denotes the regulatory ability of the *l*^th^ miRNA to the *i*^th^ target gene, *N_i_* and *O_i_* indicate the number of TFs and miRNAs binding to target gene *i* in the candidate GRN, respectively, *k_i_* is the basal expression level of the *i*^th^ gene, and *w_i_*(*n*) characterizes the stochastic noise of gene *i* for sample *n*. The model demonstrates that target gene *i* is affected by *N_i_* TFs, *O_i_* miRNAs, the basal expression level, and stochastic noise. Expression of the *i*^th^ gene in (3) can be represented by following regression form:

$$\begin{array}{ll} x_{i}\left(n\right)= & \left[y_{1}\left(n\right)\;\cdots \;y_{j-1}\left(n\right)\;y_{j+1}\left(n\right)\;\cdots \;y_{N_{i}}\left(n\right)\;-{mir}_{1}\left(n\right)\;\cdots \;-mir_{O_{i}}\left(n\right)\;1\right]\\ & \times \left[\begin{array}{c} b_{i1}\\ \vdots \\ b_{ij-1}\\ b_{ij+1}\\ \vdots \\ b_{iN_{i}}\\ c_{i1}\\ \vdots \\ c_{iO_{i}}\\ k_{i} \end{array}\right]+w_{i}\left(n\right)=\phi_{i}\left(n\right)\theta_{i}+w_{i}\left(n\right),\text{ for }i=1,2,\ldots ,N \end{array}$$ (4)

Note that Φ_i_(n) in (2) and θ_i_(n) in (4) denote the regression vector, which can be obtained from microarray and miRNA expression data. In addition, Θi in (2) and θi in (4) represent the regulatory parameter vector of the i_th_ target gene to be estimated by the least squares parameter estimation method through the real expression of genes and miRNAs.

### Construction of GENs of human aging using the system identification and system order detection methods

After constructing the regression models of the PPIN and GRN in GENs, we identified the association ability parameters *a_ij_* in (1), the TF regulatory ability parameters *b_ij_*, and the miRNA regulatory ability *c_il_* in (3) using the least squares parameter estimation method with microarray and miRNA data. All proteins in the candidate PPIN and genes in the candidate GRN were identified one protein/gene by one protein/gene. Next, the Akaike information criterion (AIC) [55] and Student's *t*-test [63] were used for determining regression model orders (i.e. *N_i_* and *O_i_*) and pruning false positive PPIs (i.e. *a_ij_*) as well as TF and miRNA regulations (i.e. *b_ij_* and *c_il_*, respectively) out of the system model order. Specifically, the insignificant parameters, *a_ij_*, *b_ij_*, and *c_il_*, out of the regression model order were deleted from candidate GENs to obtain the real GENs.

Finally, the GENs of young and elderly individuals were constructed (Figures 2a and 3a), as well as those for old women and males (Figures 4a and 5a). The functional networks of these GENs (Figures 2b, 3b, 4b, and 5b) were used to illustrate the important functional interactions of the cellular mechanisms in young and old women and males, respectively. However, further analysis is required to fully understand these GENs. Therefore, we used the PNP method based on PCA to obtain the core GEN from the corresponding GEN.

### Constructing core GENs using the PNP method

After using the AIC order detection model and Student's *t*-test to prune candidate GENs to obtain the real GENs of human aging (*p* < 0.05), we then obtained the PPIN matrix *A* and GRN matrix *B* as follows:

$$\begin{array}{l} A=\left[\begin{array}{ccccc} a_{1,1} & \cdots & a_{1,j} & \cdots & a_{1,N}\\ \vdots & \ddots & \vdots & \ddots & \vdots \\ a_{i,1} & \cdots & a_{i,j} & \cdots & a_{i,N}\\ \vdots & \ddots & \vdots & \ddots & \vdots \\ a_{N,1} & \cdots & a_{N,j} & \cdots & a_{N,N} \end{array}\right]\\ B=\left[\begin{array}{cccccccccc} b_{1,1} & \cdots & b_{1,j} & \cdots & b_{1,N} & -c_{1,1} & \cdots & -c_{1,l} & \cdots & -c_{1,O}\\ \vdots & \ddots & \vdots & \ddots & \vdots & \vdots & \ddots & \vdots & \ddots & \vdots \\ b_{i,1} & \cdots & b_{i,j} & \cdots & b_{i,N} & -c_{i,1} & \cdots & -c_{i,l} & \cdots & -c_{i,O}\\ \vdots & \ddots & \vdots & \ddots & \vdots & \vdots & \ddots & \vdots & \ddots & \vdots \\ b_{N,1} & \cdots & b_{N,j} & \cdots & b_{N,N} & -c_{N,1} & \cdots & -c_{N,l} & \cdots & -c_{N,O} \end{array}\right] \end{array}$$

where some *a_ij_*, *b_ij_*, and *c_il_*, remained while others pruned using the AIC system order detection method in the previous section were padded with zero. We then combined *A* and *B* as network matrix *H* of GENs as follows:

$$H={\left[\begin{array}{ccccccccccccccc} a_{1,1} & \cdots & a_{1,j} & \cdots & a_{1,N} & b_{1,1} & \cdots & b_{1,j} & \cdots & b_{1,N} & -c_{1,1} & \cdots & -c_{1,l} & \cdots & -c_{1,O}\\ \vdots & \ddots & \vdots & \ddots & \vdots & \vdots & \ddots & \vdots & \ddots & \vdots & \vdots & \ddots & \vdots & \ddots & \vdots \\ a_{i,1} & \cdots & a_{i,j} & \cdots & a_{i,N} & b_{i,1} & \cdots & b_{i,j} & \cdots & b_{i,N} & -c_{i,1} & \cdots & -c_{i,l} & \cdots & -c_{i,O}\\ \vdots & \ddots & \vdots & \ddots & \vdots & \vdots & \ddots & \vdots & \ddots & \vdots & \vdots & \ddots & \vdots & \ddots & \vdots \\ a_{N,1} & \cdots & a_{N,j} & \cdots & a_{N,N} & b_{N,1} & \cdots & b_{N,j} & \cdots & b_{N,N} & -c_{N,1} & \cdots & -c_{N,l} & \cdots & -c_{N,O} \end{array}\right]}^{T}$$

PCA is based on the following singular value decomposition,

$$H=U\times D\times V^{T}$$ (5)

where $U\in {ℜ}^{\left(2N+O\right)\times N},V\in {ℜ}^{N\times N}$, and *h_k_* denote the *k*^th^ row vectors of *H* for *k* = 1,…,(2*N*+*O*) i.e. H = [*h_1_*, …, *h_k_*, …., *h_2N+O_*], and the *m* column vectors of *V* are denoted as *v_m_*, for *m* = 1,…,*N*, which are defined as the right-singular vectors of *H*. The diagonal entries of the diagonal matrix, *D* = diag (*d_1_*, …, *d_m_*, …, *d_N_*), are the *N* singular values of *H* in descending order i.e. *d_1_*≥ *d_m_* ≥*d_N_*. Note that, diag (*d_1_*, *d_2_*) denotes the diagonal matrix of *d_1_* and *d_2_*, such that $\text{diag}\left(d_{1},d_{2}\right)=\left[\begin{array}{cc} d_{1} & 0\\ 0 & d_{2} \end{array}\right]$. The Eigen expression fraction (*E_m_*) is defined as

$$E_{m}=\frac{d_{m}^{2}}{\sum_{m=1}^{N}d_{m}^{2}}$$ (6)

We selected the top *M* singular vectors *v_m_* of *V* such that $\sum_{m=1}^{M}E_{m}\geq 0.85$ with the minimal *M*, so that the *M* principal components contain 85% of the GEN from an energy point of view. The projections of *H* to the top *M* singular vectors *v_m_* of *V*, or the similarities, are defined respectively as follows:

$$S\left(k,m\right)=h_{k}\times v_{m}{}^{T},\text{ for }k=1,\ldots ,\left(2N+O\right)\text{ and }m=1,\ldots ,M$$ (7)

We further defined the 2-norm distance from each gene, protein, and miRNA in GEN to the top *M* singular vectors as:

$$D\left(k\right)={\left[\sum_{m=1}^{M}{\left[S\left(k,m\right)\right]}^{2}\right]}^{1/2},\text{ for }k=1,\ldots ,\left(2N+O\right)$$ (8)

If *D*(*k*) is close to zero, it implies that the *k*^th^ gene, protein, and miRNA is independent of the top *M* singular vectors. Thus, we determine three thresholds, *th*1, *th*2, and *th*3 as shown in Figures S1 and S2 for young and elderly individuals, to respectively identify the core genes, *D*(*k*)≥*th*1 for *k* = 1,…,*N*, the core proteins, *D*(*k*)≥*th*2 for *k* = *N*+1,…,2*N*, and the core miRNAs *D*(*k*)≥*th*3 for *k* = 2*N*+1,…,*2N+O*. These were used to obtain the core GENs as shown in Figure 6a and 6b, which have the principal network structures of the GENs for young and elderly individuals, respectively.

Using the PNP method based on PCA, we obtained the core genes, proteins, miRNAs and their corresponding interactions and regulatory abilities in core GENs for young and elderly individuals. Comparing these core GENs of young and elderly individuals, part of the intersection is called the common core GEN and part of the distinction sets are called the specific core GENs. In the following section, we further explore the aging mechanisms by investigating the common core GEN and specific core GENs of elderly individuals and young controls.

## SUPPLEMENTARY MATERIALS FIGURES


