# Supplementary material for: Investigating the specific core genetic-and-epigenetic networks of cellular mechanisms involved in human aging in peripheral blood mononuclear cells
Source: Oncotarget. 2016 Feb 14;7(8):8556–79. doi: 10.18632/oncotarget.7388 (PMC4890987; doi:10.18632/oncotarget.7388)
Supplement: Supplementary file 1 [file oncotarget-07-8556-s001.pdf]

Supplemental Materials

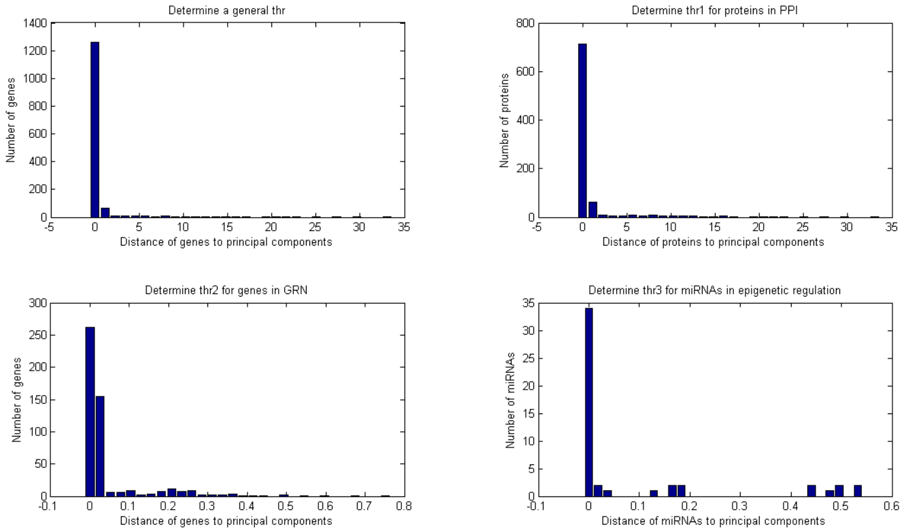

Figure S1 – The *th1*, *th2*, and *th3* for proteins, genes, and miRNAs for the PNP from the young GEN in Figure 2a to the young core GEN in Figure 6a

In the young GEN, the threshold value *th1* for proteins and TFs in PPI is 4.0, the threshold value *th2* for target genes in GRN is 0.015, and the threshold value *th3* for miRNAs in epigenetic regulation is 0.01. The vertical axis represents the number of proteins, genes, or miRNAs, whereas the horizontal axis represents the distance of proteins, genes, or miRNAs to principal components.

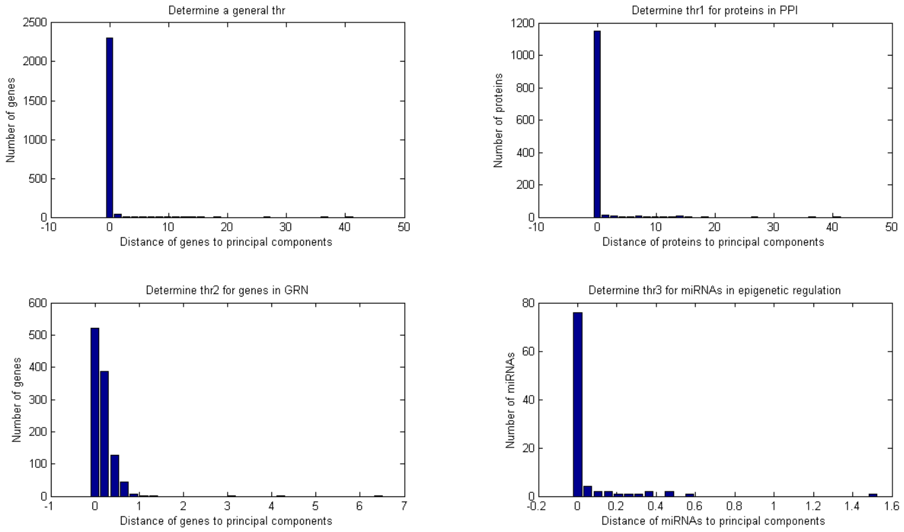

Figure S2 – The *th1*, *th2* and *th3* for proteins, genes, and miRNAs for the PNP from the elderly GEN in Figure 3a to the elderly core GEN in Figure 6b

In the elderly GEN, the threshold value *th1* for proteins and TFs in PPI is 0.6, the threshold value *th2* for target genes in GRN is 0.35, and the threshold value *th3* for miRNAs in epigenetic regulation is 0.03. The vertical axis represents the number of proteins, genes, or miRNAs, whereas the horizontal axis represents the distance of proteins, genes, or miRNAs to principal components.

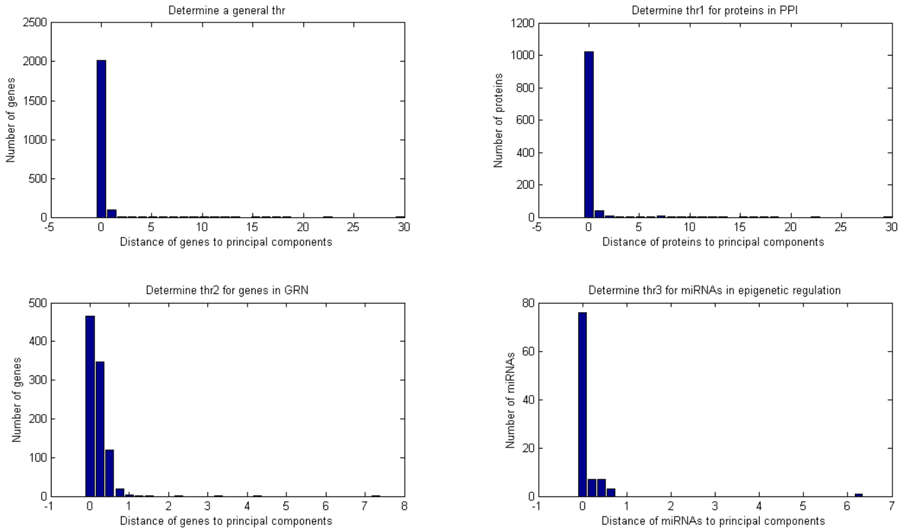

Figure S3 – The *th1*, *th2*, and *th3* for proteins, genes, and miRNAs for the PNP from the old female GEN in Figure 4a to the old female core GEN in Figure 7

In the old female GEN, the threshold value *th1* for proteins and TFs in PPI is 0.5, the threshold value *th2* for target genes in GRN is 0.4, and the threshold value *th3* for miRNAs in epigenetic regulation is 0.1. The vertical axis represents the number of proteins, genes, or miRNAs whereas the horizontal axis represents the distance from proteins, genes, or miRNAs to principal components.

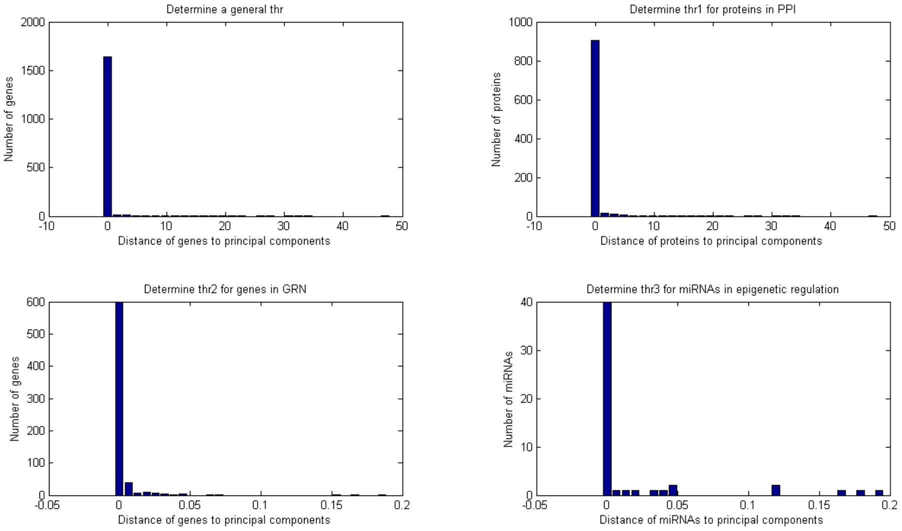

Figure S4 – The *th1, th2,* and *th3* for proteins, genes, and miRNAs for the PNP from the old male GEN in Figure 5a to the old male core GEN in Figure 8

In the old male GEN, the threshold value *th1* for proteins and TFs in PPI is 0.8, the threshold value *th2* for target genes in GRN is 0.003, and the threshold value *th3* for miRNAs in epigenetic regulation is 0.003. The vertical axis represents the number of proteins, genes, or miRNAs whereas the horizontal axis represents the distance from proteins, genes, or miRNAs to principal components.

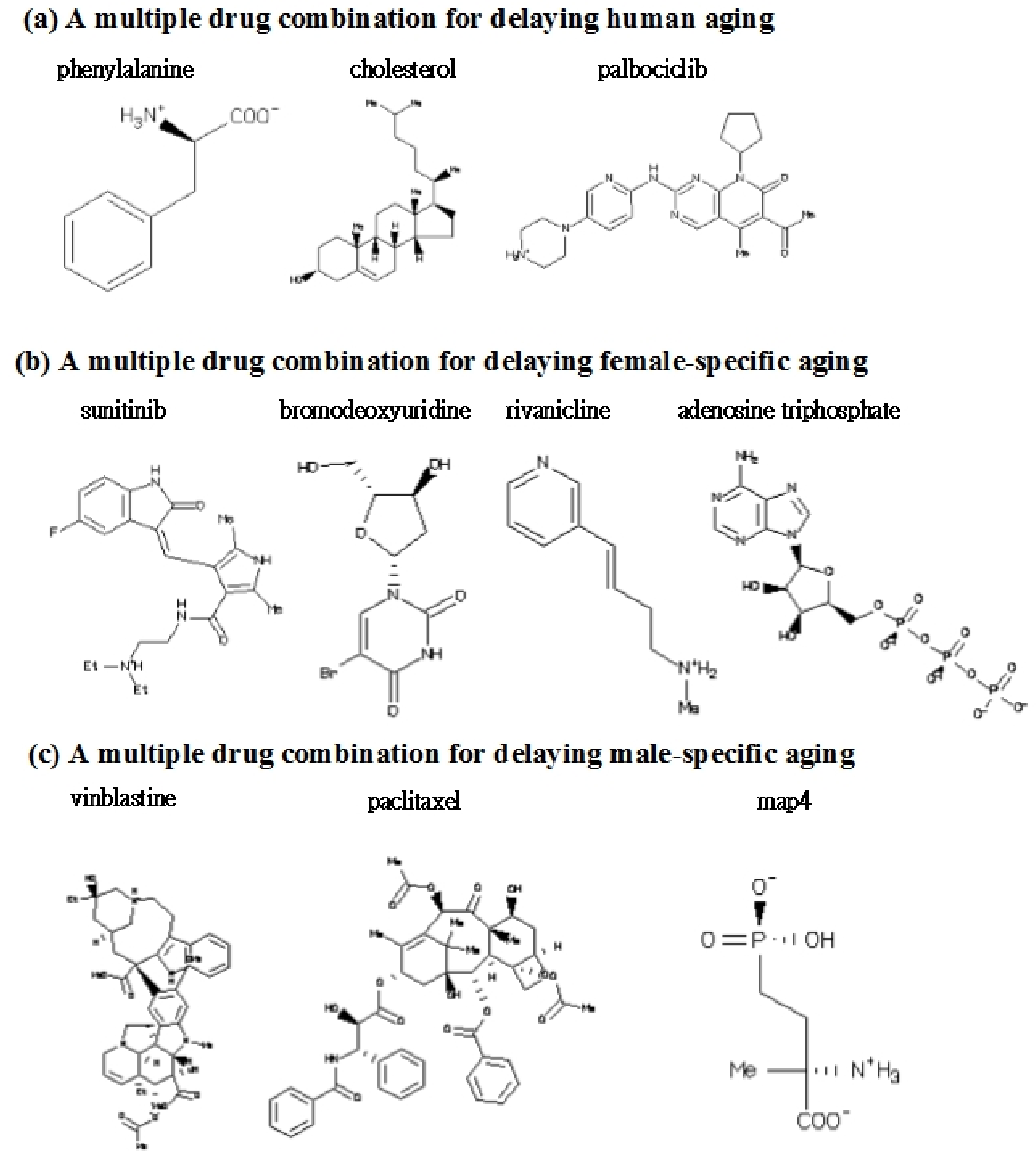

Figure S5 – Three different multiple drug combinations for delaying the aging process, the female-specific aging process, and the male-specific aging process

According to the human aging mechanisms in Figure 10, the male-specific aging mechanisms in Figure 13, and female-specific aging mechanisms in Figure 14, we obtain **three different** multiple drug combinations for delaying human aging process (a), delaying female-specific aging process (b), and delaying male-specific aging process (c).
